# Malaria mapping: understanding the global endemicity of falciparum and vivax malaria

**DOI:** 10.1186/s12916-015-0372-x

**Published:** 2015-06-12

**Authors:** Ursula Dalrymple, Bonnie Mappin, Peter W. Gething

**Affiliations:** Department of Zoology, Spatial Ecology and Epidemiology Group, University of Oxford, Tinbergen Building, Oxford, UK

**Keywords:** Malaria, Mapping, Modelling, Falciparum, Vivax, Model-based geostatistics

## Abstract

The mapping of malaria risk has a history stretching back over 100 years. The last decade, however, has seen dramatic progress in the scope, rigour and sophistication of malaria mapping such that its global distribution is now probably better understood than any other infectious disease. In this minireview we consider the main factors that have facilitated the recent proliferation of malaria risk mapping efforts and describe the most prominent global-scale endemicity mapping endeavours of recent years. We describe the diversification of malaria mapping to span a wide range of related metrics of biological and public health importance and consider prospects for the future of the science including its key role in supporting elimination efforts.

## Introduction

**Fig. 1:**
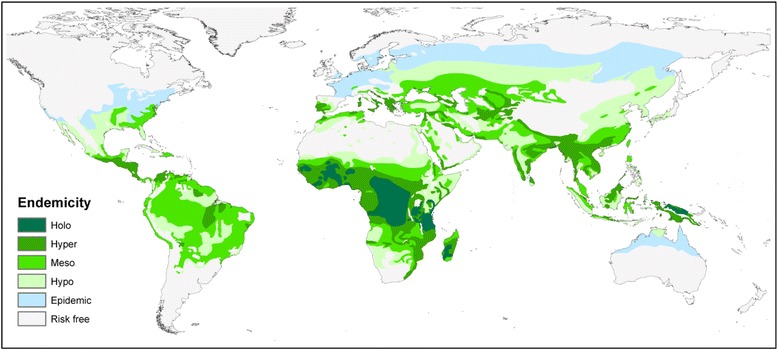
The digitised ‘Lysenko’ map of global malaria endemicity circa 1900. This historic map constructed by Lysenko and Semashko [3] during the 1960s was based on a worldwide assembly of diverse malariometric data, simple climatic rules and expert opinion. The classic strata of malaria endemicity are described, each relating to infection prevalence (parasite rate, PR) in children: hypoendemic, PR <10 %; mesoendemic, PR ≥10 % and <50 %; hyperendemic, PR ≥50 % and <75 %; and holoendemic, PR ≥75 %. This is a reproduction of the map in Hay et al. [4]

Like most vector-borne diseases, malaria endemicity is partly determined by the local environment that houses its human and anopheline hosts and mediates the interactions between them. This environmental dependency leads to complex patterns of geographical variation in malaria transmission at almost every scale. Risk is rarelyuniform whether considered between households in a village, villages in a district or districts in a country [1]. The importance of evaluating local heterogeneity has motivated a long lineage of epidemiologists and disease control practitioners to generate maps of malaria risk to better understand local disease ecology and inform control activities [2]. The first serious attempt to audit the pattern of malaria endemicity at the global scale was undertaken in 1968 by Lysenko and Semashko [3]. This represented a major synthesis of historical records, maps of various malaria metrics (such as parasite rate, vector distributions, entomological inoculation rate, sickle cell incidence) and expert opinion and yielded a global map of malaria endemicity at the assumed peak of transmission intensity around the start of the 20th century. This map, stratified into four classes of endemicity, has since been digitised [4] and remains the most plausible reconstruction of global malaria risk in the largely pre-industrial era and prior to widespread malaria control efforts [5] (Fig. 1).

It is now nearly half a century since the Lysenko map was published and, during most of that period, few efforts were made to improve on it. However, initiatives such as the continent-wide Mapping Malaria Risk in Africa/Atlas du Risque de la Malaria en Afrique (MARA/ARMA) project [6], instigated in 1997, and 8 years later the global Malaria Atlas Project (MAP) [7], catalysed a renaissance that has transformed the science of malaria risk mapping and its role in contemporary efforts to control, progressively eliminate and ultimately eradicate malaria.

In this minireview we present a condensed overview of: (i) the main factors that have facilitated the recent proliferation of malaria risk mapping efforts; (ii) prominent global-scale endemicity mapping endeavours of recent years; (iii) the diversification of malaria mapping to span a wide range of related metrics of biological and public health importance; and (iv) prospects for the future of the science including its key role in supporting elimination efforts.

### Enabling factors in the malaria mapping renaissance

#### Increasing data availability

**Fig. 2:**
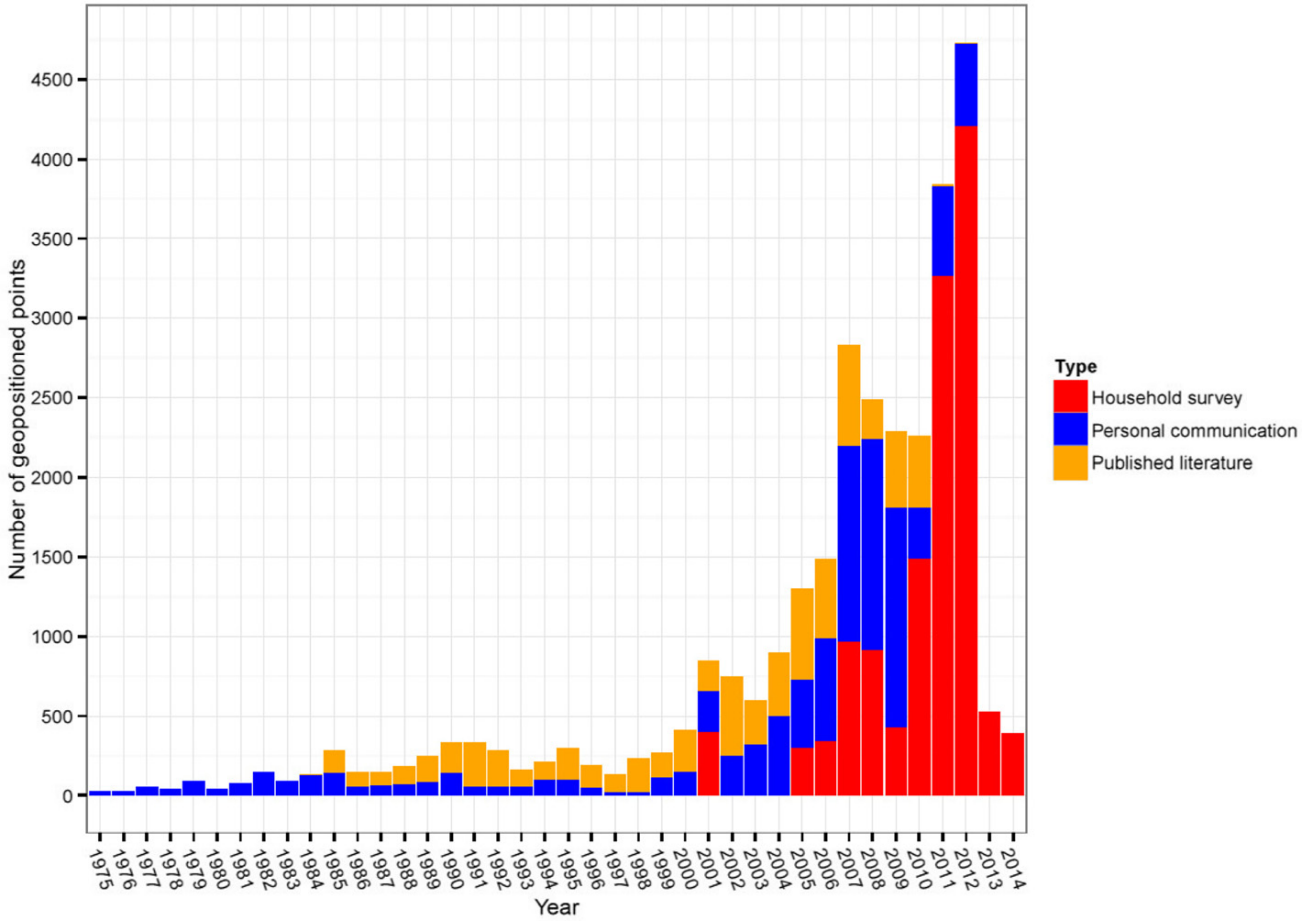
Growth in the availability of georeferenced data on *Plasmodium falciparum* parasite rate. The data shown here represent the assembly for sub-Saharan Africa maintained by the Malaria Atlas Project, with an individual ‘point’ representing a spatially and temporally unique observation of community-level infection prevalence. The search methodology used to acquire the published literature and personal communication data points is described in detail elsewhere [67], and the household survey data points are collated from the sources described above and additional reports from national malaria control programmes. The reduced numbers in 2013 and 2014 are to be expected due to the lag time between data collection and its subsequent release

Since the late 1980s, nationally representative cross-sectional household surveys have been supported by a number of multilateral initiatives including the Demographic and Health Surveys (DHS) Program [8] and the UNICEF Multiple Indicator Cluster Survey (MICS) [9]. Such surveys have frequently been conducted in malaria-endemic countries and now include a growing suite of questions designed to gauge population access and use of malaria prevention, diagnostics and treatment. Since 2006, DHS surveys have begun to obtain blood samples from children under 5 years of age (and in some surveys, pregnant women) for parasite-based diagnosis of malaria using rapid diagnostic tests (RDTs) or microscopy. Crucially for mapping, these data tend to be accompanied by geographical coordinates denoting the location of the village or community from which each individual was sampled. These standardised and prospectively designed infection prevalence (or ‘parasite rate’) data are ideally suited as a basis for national-scale endemicity mapping and have some key advantages over retrospective assemblies of *ad hoc* parasite rate data obtained, for example, from systematic literature searches. The influence of these large-scale programmes of national surveys, along with an increasing number of independent and nationally-led malaria indicator surveys, has transformed the availability of geolocated parasite rate data over the past decade (Fig. 2).

#### Improved environmental covariates of malaria risk

Along with data on malaria infection prevalence itself, a vital component of modern disease mapping methods is the inclusion of high quality data layers describing environmental or socio-demographic variables that correlate with malaria risk and can be used as empirical covariates. The ongoing refinement of satellite and airborne remote sensing platforms, and commensurate sophistication of post-processing algorithms and computational infrastructure for storage and dissemination of the resulting imagery, has led to a huge diversity of variables being included as part of spatial malaria models, as reviewed elsewhere [10]. In addition, work by malaria modelling groups has sought to modify existing geospatial variables to create malaria-specific products to enhance their utility for mapping. One recent example has been the manipulation of remotely-sensed data on land-surface temperature to create indices of temperature suitability for transmission of *Plasmodium falciparum* and *Plasmodium vivax* [11–13]. Recent work has also focused on the extension of traditionally static geospatial covariates into libraries of temporally dynamic data that potentially enable exploration of seasonal, inter-annual and long-term changes in environmental conditions on malaria transmission [10]. With the greater abundance of potential covariate layers has come an increased need for robust approaches to variable selection – allowing multivariate spatial models of malaria risk to be constructed that use an optimum set of covariates that maximise predictive power and avoid over-fitting the response data. Such approaches include Bayesian model selection procedures [14, 15] and exhaustive machine-learning techniques [10].

#### Advances in analytical techniques

The Lysenko map was constructed in the best traditions of manual cartography – with an emphasis on assimilating a wide variety of disparate data sources into a single synthesised map without any formal underlying quantitative framework. Whilst the result was an impressive summary of the state of knowledge existing at that time, this heuristic approach suffers a number of important drawbacks. Importantly, the likely accuracy of the map and how this varies from place to place can be neither measured nor communicated to end-users, placing a fundamental limitation on its use for critical public health decisions. In contrast, modern maps of malaria and other infectious diseases tend to result from formal spatial statistical models that aim to not only optimise accuracy but convey the spatially varying level of uncertainty associated with the mapped surface. The current state-of-the-art models tend to stem from a body of theory defined in the late 1990s known as model-based geostatistics (MBG) [16, 17]. MBG disease models, generally implemented in a Bayesian framework [16, 17], take point observations of disease prevalence from dispersed survey locations and generate interpolated estimates of prevalence at unsampled locations to generate continuous maps. Unlike simpler interpolation methods, MBG models capture both the inherent spatial structure displayed in a dataset (via a covariance function) and the uncertainty around that structure. They also provide a natural framework for incorporation of multivariate relationships with covariates, and the use of disease response data in continuous, count or proportion format with appropriate models for sampling error. Since such techniques were first demonstrated in a malaria mapping context [18], many useful elaborations have been developed. Gosoniu et al. [15, 19] demonstrated an approach to allow non-stationarity – enabling the spatial structure of the model to vary from place to place to better capture local variation when modelling over large areas. MBG techniques have been extended to map malaria both spatially and temporally [20], allowing data from multiple time points to contribute appropriately to a single cross-sectional map [21–23] and, more recently, to explore spatio-temporal patterns of change through time [24]. Gething et al. [25] introduced the ability to quantify aggregated uncertainty over space and time in a global-scale MBG model with use of an approximating joint simulation algorithm. This allowed predicted malaria risk levels to be summarised formally at the varying scales of geographical aggregation over which public health decisions are usually made.

### Contemporary maps of continental and global endemicity

Numerous studies have developed Bayesian geostatistical models to create national or multi-national maps of malaria risk, often intended to aid national malaria control programme policy decisions in specific regions and improve understanding of within-country patterns of spatial heterogeneity in malaria transmission and burden [26–38]. Additionally, spatial scanning methods to detect clusters (or ‘hotspots’) of intense malaria transmission at very fine spatial scales have been developed and applied at a sub-national scale. These methods can be used to identify individual homesteads within hotspots with particularly intense malaria transmission [1, 39].

**Fig. 3:**
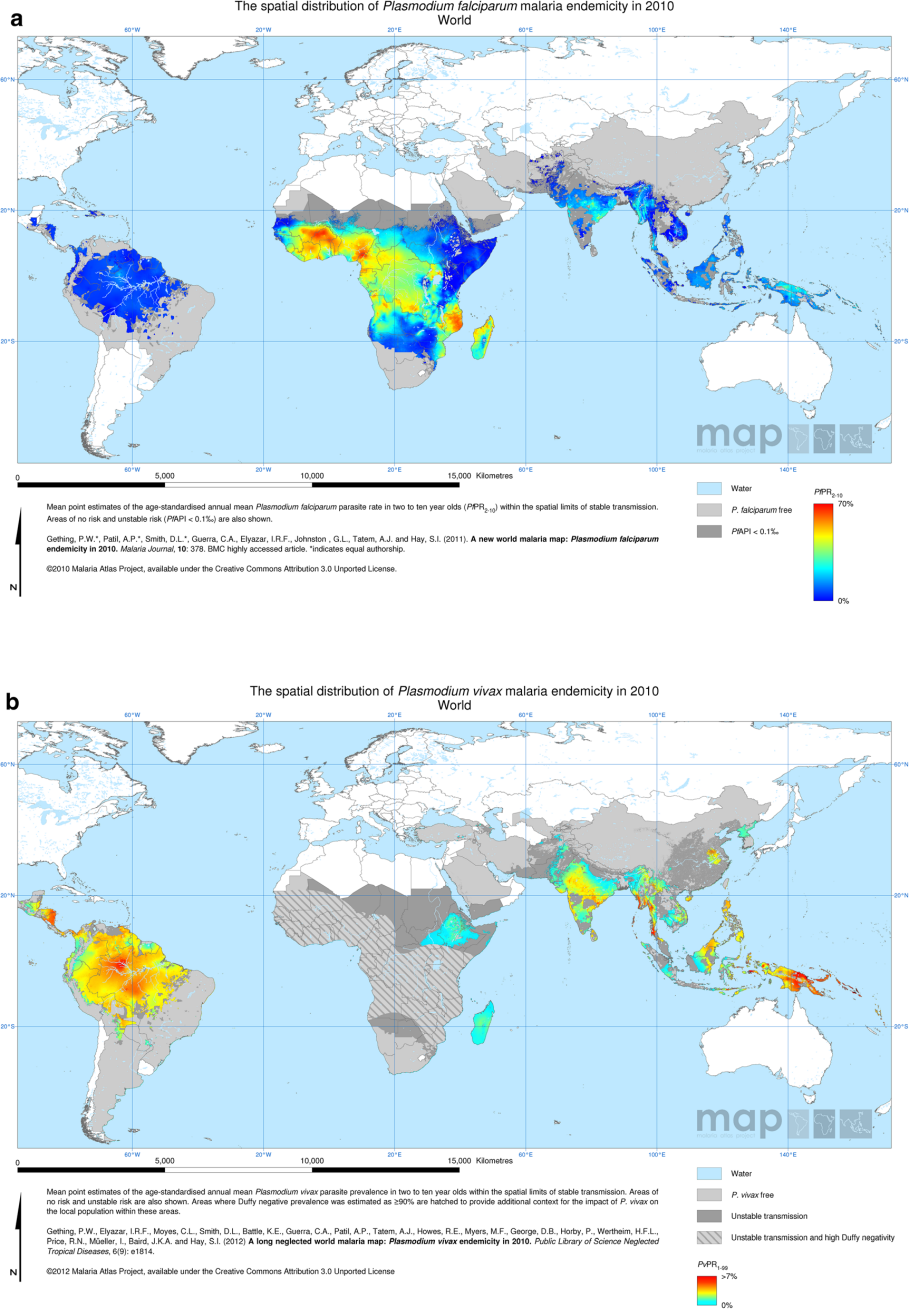
Global **a** *Plasmodium falciparum* and **b** *Plasmodium vivax* endemicity in 2010. These contemporary maps, produced by the Malaria Atlas Project, used model-based geostatistics to interpolate continuous predicted surfaces of risk based on more than 20,000 georeferenced surveys measuring infection prevalence for each parasite species. Part A is reproduced from [21] and part B is reproduced from [22]

The first attempt to map malaria endemicity at global scales using MBG techniques was completed for *P. falciparum* in 2009 by the Malaria Atlas Project [23]. This was the culmination of 5 years of data assembly [40], delineation of the limits of stable transmission [41], and methodological development to extend existing MBG approaches to incorporate additional functionality including an embedded age-prevalence standardization model [42] and the incorporation of the spherical shape of the Earth within the model computation. The resulting map, describing infection prevalence in 2–10 year olds across a 5 × 5 km resolution grid, was the first global assessment of malaria risk that used a standardised data and modelling framework and was able to provide accompanying maps describing the geographically varying uncertainty associated with each predicted pixel value. While the 2007 map marked a new era in global malaria cartography, the field continued to evolve rapidly and an updated map was generated for the year 2010 (Fig. 3a) in which, along with a large influx of new *Pf*PR surveys, some important methodological advances were made [21]. Early computational constraints in the implementation of MBG at the global scale meant that the earlier map included no environmental covariates with the exception of urbanity. For the 2010 iteration, a more efficient MCMC algorithm allowed the multivariate effects on *Pf*PR of a wider suite of 20 environmental and socio-demographic covariates to be incorporated, substantially improving predictive accuracy and the level of spatial detail that could be resolved.

In that same year, a corresponding global endemicity map of *P. vivax* prevalence was produced by the Malaria Atlas Project [22] (Fig. 3b). While the basic geostatistical architecture mirrored that developed for *P. falciparum*, the unique epidemiology [43] of this less well studied parasite species posed unique challenges for global mapping. A particular challenge was the handling of data in Africa, where a commonly held mantra is that *P. vivax* is absent due to the near-fixation in the population of Duffy negativity – a genetic blood disorder that conveys near total protection from *P. vivax* infection*.* A detailed literature review, however, demonstrated the presence of the parasite in nearly all Africa countries. Rather than branding large swathes of the continent as being vivax-free, a more elaborate approach was developed that drew from data on both *Pv*PR and population prevalence of the Duffy genetic trait [44] such that both quantities could be mapped simultaneously. These works paved the way to a comprehensive review of the global public health significance of *P. vivax*, which suggested that morbidity and fatalities caused by *P. vivax* may be substantially underestimated [45].

These maps provided new benchmark evaluations of the contemporary landscape of malaria risk worldwide. For the first time, international policy makers could draw upon a standardised methodology that allowed meaningful country-to-country comparison of malaria risk exposure for national populations. Since the publication of these 2010 maps, there has been an increasing interest in extending the methodologies to allow prediction of risk both geographically and through time, motivated in part by the need to evaluate progress towards international targets set for the year 2015 [46]. By mapping malaria risk dynamically, patterns of change can be explored. In 2014, Noor et al. produced an analysis of changing risk of *P. falciparum* malaria across Africa since 2000 [47]. This analysis used a large assembly of *Pf*PR data collected over the preceding decades, along with four static environmental covariates (precipitation, temperature suitability index, enhanced vegetation index and urbanisation) in an MBG model to generate cross-sectional risk maps for the years 2000, 2005 and 2010 [47]. This analysis allowed the first formal insights into patterns of changing malaria in Africa since the turn of the millennium, a period of major international efforts to raise funding and scale up control efforts. The study described evidence of declining *Pf*PR in nearly all endemic Africa countries between 2000 and 2010, but in many cases these declines were relatively modest.

A more recent study by the Malaria Atlas Project has also sought to evaluate changing risk patterns in Africa, for the more recent year of 2015, with some important differences in input data and approach (Bhatt S, Weiss DJ, Mappin B, Dalrymple U, Cameron E, Bisanzio D, et al: Insecticide-treated nets (ITNs) in Africa 2000–2017: coverage, system efficiency and future needs to achieve international targets, unpublished). Most significant was the development and incorporation of detailed spatio-temporal reconstructions of coverage patterns for the major malaria control interventions over the same time period [24]: insecticide-treated bed nets (ITNs); indoor residual spraying (IRS); and artemisinin-based combination therapy (ACT) antimalarials for malaria case management. Incorporating these within a space-time MBG framework allowed improved estimates of infection prevalence through time. By also including a wide range of temporally dynamic background covariates since 2000 [10], it was possible to disentangle the relative contributions of each intervention to the observed declines in *Pf*PR, in the context of any changes in underlying environmental conditions. This work provided important evidence on the impact of large-scale control efforts in Africa since the turn of the Millennium.

### Beyond prevalence: the diversification of malaria mapping

#### Deriving malariometrics from parasite rate estimates

The developments in mapping malaria infection prevalence, described above, have spawned a larger and more diverse body of work, allowing maps of parasite rate to be used as an input to predict the distribution of a wide range of other malariometrics with distinct utility to biologists, epidemiologists and decision-makers. These have included the mapping of clinical incidence rates, entomological inoculation rates (EIRs), the basic reproductive number (*R*) and the burden of morbidity due to malaria in pregnancy.

The measurement of malaria incidence (the number of clinical cases that occur annually within a given population) is typically measured by one of two approaches: either by using direct data on observed cases detected via routine surveillance systems; or by using maps of infection prevalence and using a model to convert this metric into a plausible value of clinical incidence at each mapped location (known as the ‘cartographic’ approach). In much of sub-Saharan Africa, and in particular those countries with the higher malaria burdens, routine surveillance data are not considered sufficiently robust to use as a basis for estimating clinical incidence or evaluating trends through time [24]. The development of continuous parasite rate maps has made it possible to model statistically the relationship between *P. falciparum* prevalence and clinical incidence rates. Initial efforts to construct a *Pf*PR-incidence relationship for *P. falciparum* burden estimation used data-driven fits with varying sophistication from first-order stratification by endemicity class to hierarchical Gaussian process regression [48–50], and projections based on the calibration of a steady-state compartmental transmission model [51]. In 2015, Cameron et al. used three of the most contemporary published prevalence-incidence models were calibrated against a purpose-built dataset of incidence counts from numerous sites across sub-Saharan Africa (Cameron E, Battle KE, Bhatt S, Weiss DJ, Bisanzio D, Dalrymple U, et al.: Defining the relationship between infection prevalence and clinical incidence of Plasmodium falciparum malaria: an ensemble model, Submitted). The combined predictive power of this ensemble model allowed forecasts of expected malaria incidence with limited uncertainty, and highlighted general conceptual agreement between the models*.* The ensemble model has since been utilised, alongside the Malaria Atlas Project’s estimations of yearly *Pf*PR, to estimate the changing incidence of *P. falciparum* malaria from 2000 to 2015.

The EIR, or entomological inoculation rate, describes the number of expected bites from infected mosquitoes per person per unit time and is often used as a standardised measure of transmission intensity [21]. Work has been done to assemble observations of EIR across Africa and define their relationship with *Pf*PR [52]. In an analogous way to the cartographic estimation of clinical incidence, this has allowed maps of infection prevalence to be converted into maps of EIR, describing this key entomological quantity geographically across the endemic world [21]. The same work also included a model to extend *Pf*PR maps to describe the global distribution of the basic reproductive number, *R*, for *P. falciparum* malaria. *R* quantifies the potential of *P. falciparum* to spread throughout a population (formally the number of new cases arising per index case per generation of the parasite) and provides important insights into, for example, the magnitude of impact that control efforts must have at each location in order to drive transmission towards elimination.

Estimates of the number of pregnant women at risk of malaria infection globally have been made [53] by combining national estimates of numbers of pregnancies for 2007 and MAP’s 2007 and 2003 estimates of global *P. falciparum* [41] and *P. vivax* endemicity [54], respectively. Although the World Health Organization (WHO) estimates annually the number of pregnant women at risk of malaria in Africa, this study provided the first comprehensive and contemporary estimation of the number of pregnancies at risk of malaria outside of Africa.

## Mapping for elimination

Long-term international policy around malaria control is increasingly reoriented to achieve progressive elimination of malaria country-by-country with the ultimate goal of reaching eradication of the disease [55, 56]. An initial utility of global endemicity maps in this context has been as one component of a wider assessment of relative elimination feasibility between countries, helping guide prioritisation and target-setting [57]. As more malaria-endemic countries enter the elimination phase, new challenges arise for malaria cartography to provide geospatial information tailored to the distinct operational requirements of elimination activities. An immediate technical challenge arises from the difficulty in obtaining useful metrics of malaria transmission at very low levels of transmission. Traditional parasite rate surveys become underpowered to detect very rare infections, and research is underway to examine a range of alternative metrics for mapping, including molecular-based parasite detection or identification of serological markers of infection exposure [58–60]. In elimination scenarios, the diagnostic accuracy of response data becomes more important in order to detect subpatent infections which are thought to account for 20–50 % of human-to-mosquito transmissions in low endemicity areas [61]. Investment in more sensitive case detection methods is required to accurately assess transmission intensity [62]. Additionally, methods to standardise diagnostic data inputs prior to mapping are required to eliminate uncertainty, especially in elimination areas. Regression models have been developed in recent years between both microscopy and PCR [61], and RDT and microscopy [63]. These models can be applied to observed prevalence measured by one diagnostic test in an elimination area to estimate the expected observed prevalence using an alternative method of diagnosis. Additionally, measuring progress towards elimination is aided by the ever-increasing availability of map data and measurements of parasite rate over time and space which can be used for comparison.

Further challenges in defining geographic patterns of risk arise from the issue of human movement. When cases become rare, the relative contribution of imported malaria – infections originating outside the eliminating country – tends to increase until they can become the primary reason for transmission being sustained. This complicates the description of risk patterns and necessitates an understanding of human movement alongside data on observed infections. In a study in Namibia, Tatem et al. integrated mobile phone data (which can serve as a proxy for human movement patterns) with case-based risk maps to predict hotspots of transmission in generally low-transmission settings [64]. Other work has sought to use a range of input metrics and mapping techniques to identify the fine-scale or seasonal variations in risk which become important in understanding the highly heterogeneous pattern of risk in elimination settings [65, 66].

## Conclusions

This review has summarised the evolution of malaria risk mapping over the past decade and the improvements in data availability, computational power and methodological developments that have facilitated it. This ongoing development has transformed malaria risk mapping from an art to a science, and can now bring mature and statistically robust approaches to bear on a diverse range of cartographic questions. As the global malaria landscape continues to change over the coming years, these geospatial approaches must continue to evolve in order to provide accurate descriptions of change, insight into the many factors driving those changes and, ultimately, to continue to contribute to evidence-based malaria control and elimination activities worldwide.

## Abbreviations

ACT: Artemisinin-based combination therapy
DHS: Demographic and Health Surveys
EIR: Entomological inoculation rate
IRS: Indoor residual spraying
ITN: Insecticide-treated bed net
MAP: Malaria Atlas Project
MARA/ARMA: Mapping Malaria Risk in Africa/Atlas du Risque de la Malaria en Afrique
MBG: Model-based geostatistics
MICS: Multiple Indicator Cluster Survey
PCR: Polymerase chain reaction
PR: Parasite rate
RDT: Rapid diagnostic test
WHO: World Health Organization
